# Null model analyses of temporal patterns of bird assemblages and their foraging guilds revealed the predominance of positive and random associations

**DOI:** 10.1002/ece3.5372

**Published:** 2019-06-20

**Authors:** Martin Korňan, Marek Svitok, Anton Krištín

**Affiliations:** ^1^ Department of Applied Zoology and Wildlife Management, Faculty of Forestry Technical University in Zvolen Zvolen Slovakia; ^2^ Centre for Ecological Studies Veľké Rovné Slovakia; ^3^ Department of Biology and General Ecology, Faculty of Ecology and Environmental Sciences Technical University in Zvolen Zvolen Slovakia; ^4^ Department of Ecosystem Biology, Faculty of Science University of South Bohemia České Budějovice Czech Republic; ^5^ Institute of Forest Ecology Slovak Academy of Sciences Zvolen Slovakia

**Keywords:** birds, community ecology, compensatory dynamics, co‐occurrence indices, effects of matrix properties, species association

## Abstract

Patterns of species associations have been commonly used to infer interactions among species. If species positively co‐occur, they may form predominantly neutral assemblages, and such patterns suggest a relatively weak role for compensatory dynamics. The main objective of this study was to test this prediction on temporal samples of bird assemblages (*n* = 19, 10–57 years) by the presence/absence and quantitative null models on assemblage and guild levels. These null model outcomes were further analyzed to evaluate the effects of various data set characteristics on the outcomes of the null models. The analysis of two binary null models in combination with three association indices revealed 20% with significant aggregations, 61% with random associations, and only 19% with significant segregations (*n* = 95 simulations). The results of the quantitative null model simulations detected more none‐random associations: 61% aggregations, 6% random associations, and 33% segregations (*n* = 114 simulations). Similarly, quantitative analyses on guild levels showed 58% aggregations, 20% segregations, and 22% random associations (*n* = 450 simulations). Bayesian GLMs detected that the outcomes of the binary and quantitative null models applied to the assemblage analyses were significantly related to census plot size, whereas the outcomes of the quantitative analyses were also related to the mean population densities of species in the data matrices. In guild‐level analyses, only 9% of the GLMs showed a significant influence of matrix properties (plot size, matrix size, species richness, and mean species population densities) on the null model outcomes. The results did not show the prevalence of negative associations that would have supported compensatory dynamics. Instead, we assume that a similar response of the majority of species to climate‐driven and stochastic factors may be responsible for the revealed predominance of positive associations.

## 1 INTRODUCTION

Compensatory dynamics are believed to play an important role in community organization and functioning especially under environmental stress (Gonzalez & Loreau, 2009). It involves repeated phases of population growth and decline among species in response to continuous environmental pulses, where pulses represent high‐frequency environmental fluctuations (Gonzalez & Loreau, 2009). Repeated phases of population growth and decline of species are also characteristic for correlated dynamics depending on fluctuating resources (e.g., Clotfelter et al., 2007; Enemar, Sjöstrand, Anderson, & Proschwitz, 2004; Holmes, Sherry, & Sturges, 1986), but these are primarily climate‐driven neutral community processes rather than competition. The concept of compensatory dynamics came from the notion of density compensation in island faunas by MacArthur, Diamond, and Karr (1972). This concept was originally understood as part of competition theory as a process driving temporal fluctuations of species within communities.

The theory of compensatory dynamics assumes that if competitive interactions are important in driving year‐to‐year fluctuations in abundance, then changes in the abundance of one species should be generally accompanied by compensatory changes in the abundances of other members of community (Houlahan et al., 2007). However, based on meta‐analyses of 41 data matrices from various taxonomic groups except birds, Houlahan et al. (2007) concluded that compensatory dynamics are rare in natural ecological communities. Korňan and Svitok (2018) tested this concept on 19 long‐term data sets of bird assemblages, and their pairwise null model analyses led to similar conclusions. Testing the general applicability of the compensatory dynamics concept in natural communities showed more contradictory than supporting conclusions (Gonzalez & Loreau, 2009; Houlahan et al., 2007; Korňan & Kropil, 2014b; Korňan & Svitok, 2018). A predominance of correlated and random dynamics can indicate a common response of the majority of species within communities to climate and stochastic drivers of community dynamics rather than competition.

In our current study, we test the general applicability of the concept of compensatory dynamics in bird assemblages. Up to now, world‐wide meta‐analyses of temporal patterns of bird assemblages testing this concept have not been published in the ecological literature (see reviews Dhondt, 2012; Mikusiński et al., 2018; Wiens, 1989a) except our previous study based on interactions among individual species pairs (Korňan & Svitok, 2018). Here, we go beyond pairwise relationships and focus on assembly processes at community and foraging guild levels with a focus on both qualitative and quantitative data.

Testing whether communities are driven by compensatory, correlated, or random dynamics can be based on analyses of species associations (Houlahan et al., 2007; Korňan & Svitok, 2018; Schluter, 1984). Negative species associations (segregations) may indicate compensatory dynamics, positive species associations (aggregations) correlated dynamics and random associations random dynamics. We here applied null model analysis to test for nonrandom species associations. Null model analysis is a frequently used tool in searching for species associations (co‐occurrence) patterns in spatial and temporal data sets (Gotelli & McCabe, 2002; Gotelli & Ulrich, 2010; Korňan & Svitok, 2018; Ulrich & Gotelli, 2010). Gotelli and Graves (1996) and Gotelli and Ulrich (2012) defined null model analysis as a pattern‐generating model that is based on randomization of ecological data or random sampling from a known or specified distribution. Certain elements of the data are held constant, and others are allowed to vary stochastically to create a random assemblage pattern. The randomization is designed to produce a pattern that would be expected in the absence of a particular ecological mechanism. Null models are used in combination with various binary or quantitative species association indices, of which the checkerboard score (Stone & Roberts, 1990) is probably the most popular.

Analyses of species associations are commonly done on community‐level matrices in which a community‐level species association index is computed for all species combined. Such analyses may suffer from dilution effects (Diamond & Gilpin, 1982). This effect is expected because species from various guilds are combined in the analysis of a community‐level matrix, which produces diluted index values by covering noncompeting species and could bias the results. Therefore, guild‐level analysis is needed to overcome these problems.

Guilds are understood as basic structural units of communities sensu Root (1967). In the original “Rootian” sense, guilds were defined as a group of species that exploit the same class of environmental resources in a similar way. Environmental resources may include diet, foraging substrates, and breeding sites. Due to similar niche requirements among guild members, a higher level of interspecific competition is assumed than among all members or random species groups of assemblages (Blondel, 2003; Korňan & Kropil, 2014a; Mac Nally, 1983; Simberloff & Dayan, 1991). Consequently, guilds can be used as natural species groupings for studies of species associations to test assumptions of competition theory.

In our previous study using pairwise null model analysis of species association on 19 long‐term data sets (>10 years) of bird assemblages, we detected the overall very low frequency of significant species pairs with a strong predominance of positive associations (Korňan & Svitok, 2018). In this study, with the same 19 long‐term data sets of bird assemblages, we tested species associations at higher hierarchical levels (guilds and assemblages). Having conducted analyses of three basic levels of community organization (species pairs, guilds, and whole assemblage), we can develop much stronger interferences about the general applicability of the compensatory dynamic concept for bird assemblages. This is the first such study testing species associations in temporal bird data sets. In addition, we tried to test effects of matrix properties and null model setting on the null model outcomes and thus enhance generality of our conclusions and target possible weaknesses in the results. In particular, we (a) examined the frequency of different species association patterns revealed by null model simulations (aggregation, random, segregation) and (b) assessed the relationships between various data set characteristics (such as matrix size, duration of study, and proportion of zero in a matrix) and the outcomes of the null model simulations.

## 2 MATERIAL AND METHODS

### 2.1 Sources of data

We used the scientific reference and citation databases Clarivate Analytics Web of Science and SCOPUS for an extensive global search for long‐term bird assemblage studies in which data sets were published. We also searched for this type of studies in cited references in scientific papers on the topic. We only selected studies in which the mapping method as a bird census technique was applied. Mapping method is a standard census technique primarily designed for estimating abundances of territorial and noncolonial birds in a study plot (Bibby, Burgess, Hill, & Mustoe, 2000; IBCC, 1969; Williams, 1936). As a selection procedure, the minimum census plot size requirement was 7 ha. In total, we found 19 long‐term studies (≥10 years) of this type from three habitat types: 15 forests, 3 open habitats, and 1 city park (Appendix S1). Time periods of censuses ranged from 10 to 57 years while the studies spanned the period from 1927 to 2014. Species richness of the plots across census periods ranged from 14 to 78 species. Ten of the forest plots had close to primeval character, and five were second growth. Close to primeval forests (pristine, virgin, old‐growth) are primary forests with the original composition of plant communities that have never been cut and can be only negatively affected by atmospheric pollution or overgrazing by game. Seventeen studies were conducted in Europe, and two studies in North America. Details and literature sources of the data sets are given in Appendix S1.

### 2.2 Preparation of data matrices

Published data sets were organized as quantitative (abundance) data matrices in which each row represents a species and each column represents a year. Population abundances from mapping method estimates are expressed as the number of occupied territories on a census plot. Each territory is equivalent to one breeding pair; therefore, breeding pairs or territories have exactly the same meaning and can be used interchangeably. In both binary and quantitative matrices, all species within bird assemblages were included (Appendix S1). For example, the densities of some species (e.g., some woodpeckers, corvids, owls, birds of prey) that have very large territories cannot be estimated on small study plots and were marked “+” in the source papers as breeding species with tracing densities which means that densities of these species are impossible to estimate due to their very low abundance, but their abundance is lower than 0.5 territories per census plot. These species are called tracing species and their population densities tracing densities. Because we did not want to lose the information on tracing species, we added small assumed density value constants (0.1 p/10 ha for woodpeckers and smaller passerines; 0.05 p/10 ha for smaller raptors and corvids; 0.004 p/10 ha for large raptors) to all year presences that varied among species, reflecting their assumed population densities in a habitat.

To analyze foraging guilds, we classified all species from all assemblages into guild categories by means of an *a priori* approach (see below and Appendix S2). Species classifications of all European and North American birds in matrices can be found in Appendix S2. Foraging guild matrices also included species with tracing densities by adding density values constants exactly as in case of whole assemblage matrices.

For each data set, we measured several characteristics that could influence the results of the null model simulations. Those characteristics were the size of the census plots (area in hectares, spatial extent), the size of the matrix (number of rows × number of columns), the duration of the study in years, the total number of species, the proportion of zeroes that were in the matrix, mean species density, and coefficient of variation (CV) of species density.

### 2.3 Classification of foraging guilds

We applied an *a priori* approach (Wiens, 1989a) to classify European and North American birds into foraging guilds. We used number of published papers and monographs with descriptions of feeding tactics, diet niches, and use of foraging substrates (e.g., Reif, Hořák, Krištín, Kopsová, & Devictor, 2016). We based detailed classification of individual bird species on the dominant feeding strategy during the breeding period, and it is given in the Appendix S2. The basic criteria of how birds were classified are as follows:

Insectivorous birds: primary diet consists of invertebrates (>50%); less important could be also other food types, for example, seeds, berries, and leaves. Insectivorous birds were further divided into the following guilds, considering >50% of time spent by feeding on a particular substrate or feeding on a particular dietary type:

1. Trunk foragers: species taking invertebrate prey mainly on tree trunks, less commonly larger branches as foraging substrates. Bark gleaners (creepers, nuthatches) and trunk probers (woodpeckers) cover this foraging guild.
2. Foliage foragers: the most species diverse guild in forest habitats. Species prey on invertebrates (caterpillars, flies, butterflies, beetles, etc.) from leaves, twigs, or smaller branches, using various prey‐capture strategies as gleaning, hovering, and snatching. Among typical foliage gleaners are tits, sylviid warblers, icteriid warblers, phylloscopiid warblers, crests, etc.
3. Aerial foragers: species preying on invertebrates mainly by sally, sweep, or hawk tactics from air and less frequently from other types. Some species may use arboreal substrates in relatively high frequency, for example genus *Ficedula*, for example during caterpillar or aphid outbreaks (Krištín, 1992). Members of this guild are muscicapiid flycatchers, swifts, martins, and swallows.
4. Ground foragers: species foraging in the lower strata such as litter of herb layer. Typical species of this guild are pipits, larks, thrushes, accentors, etc.

Nectar and sap feeders: foraging strategy typical for the North American hummingbirds. The birds typically suck nectar from flowers and/ or oozing sap (including attracted invertebrates) from tree holes drilled by woodpeckers.

Omnivorous guild: species feeding on various types of resource (living or dead invertebrates, vertebrates, plants) regardless of vegetation layer. This foraging category is typical of most corvids.

Plant and seed eaters: species primarily consuming vegetative parts (>50%, e.g., seeds, flowers, leaves, fruits, and berries) in any place in the habitat and less frequently invertebrates. Typical plant eaters are pigeons, doves, finches, crossbills, etc.

Raptors: species feeding mainly on vertebrates and carrion (>50%), less frequently on invertebrates. Foraging strategies in this guild were not taken into consideration. Typical raptors are owls and birds of prey.

Water foragers: species feeding mainly on water invertebrates (>50%) from water bodies, the surface or bottom of lakes, or streams. Typical water foragers are ducks, wagtails, and dippers.

### 2.4 Statistical analyses

#### 2.4.1 Species association indices

##### Binary (presence/absence) indices

To test species co‐occurrence patterns in long‐term data sets in the simplest form, three binary indices were used: number of checkerboards (CHECKER), checkerboard score (C‐score), and variance ratio (V‐ratio). The checkerboard index counts the number of species pairs in the data matrix that form perfect checkerboards (Diamond, 1975; Gotelli, 2000). If interspecific competition drives community dynamics, we expect to find significantly more species forming checkerboards in a real data matrix than in simulated random data matrices by null models. The second index used was the checkerboard score (Gotelli, 2000; Stone & Roberts, 1990). This index is related to the former index and measures the degree to which species co‐occur but does not require perfect segregation between species. The C‐score represents the mean number of checkerboards per species pair in the community. For a community structured by competitive interactions, we should expect C‐score values to be significantly higher than in random assemblages of species. The last index applied for testing species co‐occurrences was the variance ratio (Gotelli, 2000; Robson, 1972; Schluter, 1984). This index is based on the computation of the ratio of the variance in the total number of species in year samples to the sum of the variances of the individual species (Schluter, 1984). If the assumption of interspecific competition is valid, the observed index value should be significantly lower than in the simulated data matrices under null models.

##### Quantitative indices

To test for species covariance in quantitative data matrices, three quantitative indices were used in null model analyses–the quantitative number of checkerboards (CA_ST_), the quantitative number of aggregations (AA_ST_), and Chaoʼs index of similarity for *n* communities (MA). The quantitative number of checkerboards index is the analogue of the presence/absence checkerboard index with abundance or density data. The index is a count of the total number of abundance checkerboards in 2 × 2 species‐by‐year or species‐by‐site submatrices in the matrix (Ulrich & Gotelli, 2010). The standardized index values range from 0.0 to 1.0, with high CA values indicating more negative covariation in a matrix (Ulrich & Gotelli, 2010). An assemblage in which interspecific competition operates should have significantly higher values of the CA index than would be expected under the null model. The second applied metric for measuring quantitative species associations was the number of quantitative aggregations. This index is a count of the aggregated 2 × 2 species‐by‐year or species‐by‐site submatrices in the matrix (Ulrich & Gotelli, 2010). The standardized value of the AA index can range from 0.0 to 1.0, with high values of AA indicating positive covariation in the abundance of species (Ulrich & Gotelli, 2010). The index value in communities controlled by interspecific competition should be significantly lower than in a random assemblage of species. The third metric used was Chaoʼs index (Chao, Jost, Chiang, Jiang, & Chazdon, 2008), which was developed as an extension of the Morisita index of similarity for two communities to a matrix‐wide metric for *n* communities. The index can reach values from 0.0 to 1.0, with low values indicating dissimilarity of year samples that is interpreted as a measure of negative species association, that is, low values of the MA index indicate communities driven by competition processes.

#### 2.4.2 Null model algorithms

Nine binary null models have been used in ecological studies to analyze the presence/absence data matrices (Gotelli, 2000). These null models ranged from the most liberal (row and column equiprobable constraint) to the most conservative (sums of rows and columns fixed constrain) solutions in null model analysis and their combinations. Among these binary models, two models with the best statistical properties in terms of Type I and II errors were applied–SIM2 (row sums fixed and column equiprobable constraint) and SIM9 (sums of rows and columns fixed constraint) (Gotelli, 2000). The algorithm SIM9 was not applicable for simulations by V‐ratio index because the constraint does not enable the production of random variance of rows and columns in the null model simulations. The SIM2 algorithm treats the occurrence of each species in each year as proportional to the total number of species in that year. Therefore, the species number in each year will vary somewhat from one simulation to the next, although the relative rankings of years in species richness will be maintained on average (Gotelli & Graves, 1996). Treating years as equiprobable is a reasonable assumption because all year samples were taken in the same sites with very similar environmental conditions in most ecosystems (climax forests). Climate could vary to some extent from year to year, but the floristics and structure of the forest stands remained very similar. The SIM9 algorithm fixes row and column totals, which preserves the probability of occurrence of species among years and the probabilities of occurrence among species. For time series data, SIM9 implies that some time periods have a greater suitability for the occurrence of all species or that some time periods have higher detection probabilities of some species than others. For details on model properties, see Gotelli (2000).

In contrast to binary data, quantitative data matrices offer a much wider range of solutions for the randomization of data matrices by null models. Quantitative null models do not have as long of a history as binary models, and most of them were developed in ecology in recent years (Ulrich & Gotelli, 2010). We used the same statistical criteria as described above for the selection of quantitative null model algorithms. Based on extensive diagnostic tests by Ulrich and Gotelli (2010), IT (rc) and IA (aa) algorithms were used here as the optimal solution for quantitative null models. Algorithm IT assigns individuals randomly to matrix cells with probabilities proportional to observed row and column abundance totals until, for each row and column, total abundances are reached (Ulrich & Gotelli, 2010). Algorithm IA reassigns all individuals randomly to matrix cells with probabilities proportional to observed row and column abundance totals until the matrix‐wide total number of individuals is reached (Ulrich & Gotelli, 2010).

We applied a pluralistic analytical approach and tried to evaluate the effect of two binary and quantitative null models with acceptable statistical and ecological assumptions in combination with three commonly used alternative indices for testing species associations. Our attempt was to show that different views on null model analyses may alter the conclusions to some extent. We consider this pluralistic approach of using several models and indices to be more objective, as it offers several views on null model analyses of species associations which can be methodologically acceptable and which do not necessarily lead to the same conclusions. Using a single null model in combination with an index would yield “unambiguous” results, but may produce a biased view of the problem.

#### 2.4.3 Null model analyses

Binary null model analyses were performed in the numerical package EcoSim 7.0 (Gotelli & Entsminger, 2001), and quantitative null model analyses were performed in Turnover 1.1 (Ulrich, 2010). A sequential swap randomization algorithm was used for randomizations of the original data matrices in the binary null models, since the thorough evaluation of this algorithm demonstrated its good statistical properties and performance (Gotelli & Entsminger, 2003).

The association index was calculated for each simulated matrix, and the statistical significance of the observed matrix was calculated as the frequency of simulated matrices that had indices that were equal to or were more extreme than the observed index. Since we were interested in the variance of these indices to both sides, we searched for segregation as well as for aggregation patterns in species distributions. Two‐tailed tests were used to test for the significance of the observed index values for the binary models and the one‐tailed test for quantitative models due to software presets. The tail probabilities were calculated from null model distributions based on 10,000 simulations, and the significance level was set to *α* = 5%.

#### 2.4.4 Analysis of the null model outcomes

The results of null model analyses were classified into three possible categories of outcomes: aggregation (co‐occurrence of species significantly higher than in a random assemblage), segregation (co‐occurrence of species significantly lower than in a random assemblage), and random pattern (nonsignificant result of a null model simulation). These null model outcomes were further analyzed in order (a) to assess whether the guilds show different patterns of community assembly, that is, whether the null model outcomes differ among guilds and (b) to evaluate the relationships between various data set characteristics and the outcomes of the null models.

The differences in the null model outcomes among guilds were assessed using generalized mixed effect models (GLMM) with individual studies (data matrices) as a random grouping factor. Since some guilds were (nearly) exclusively associated with only one type of null model outcome, we faced (quasi) complete separation problems which caused that maximum likelihood estimation to fail to converge (Allison, 2003). The separation problems were handled by specifying weakly informative priors in Bayesian GLMMs (Hadfield, 2018). We used a half‐Cauchy distribution with a scale parameter of 25, a prior distribution that was recommended as a default tool to handle complete separation in binomial models (Gelman, 2006; Gelman, Jakulin, Pittau, & Su, 2008). Markov Chain Monte Carlo (MCMC) algorithm was used to approximate the posterior distribution of model parameters (McCarthy, 2007). Markov Chain Monte Carlo generates a series of values from a parameter space in which each value is conditional on the previous number. We used a Markov chain length of 10 million iterations. Since each MCMC sample depends on the value of the previous sample, successive values may be correlated. Correlation in the Markov chain impairs efficiency of the sampling algorithm, and therefore, we reduced the dependency in two ways (Hadfield, 2018; McCarthy, 2007): (a) we discarded the initial 10,000 samples (burn‐in) since the first iterations usually show a strong dependency on the starting parametrization, and (b) we saved every 5,000th sample (thinning) to reduce autocorrelation. The setup of burn‐in and thinning interval led to posterior distributions of 1,998 samples for all parameters. The convergence (dependence on the starting parametrization) and mixing of chains (diminishing the correlation) were assessed by MCMC trace plots and by examining autocorrelation among posterior samples. In case of poor mixing, the length of the chain was increased to 100 million iterations. In the final models, all estimated parameters showed autocorrelations <0.05. We reported differences in the deviance information criteria between each model and its associated null model (ΔDIC = DIC*_m_* − DIC_0_) and considered the model to be statistically significant if the 95% highest posterior density interval (credible interval) of fixed effect estimates did not span zero.

**Figure 1:**
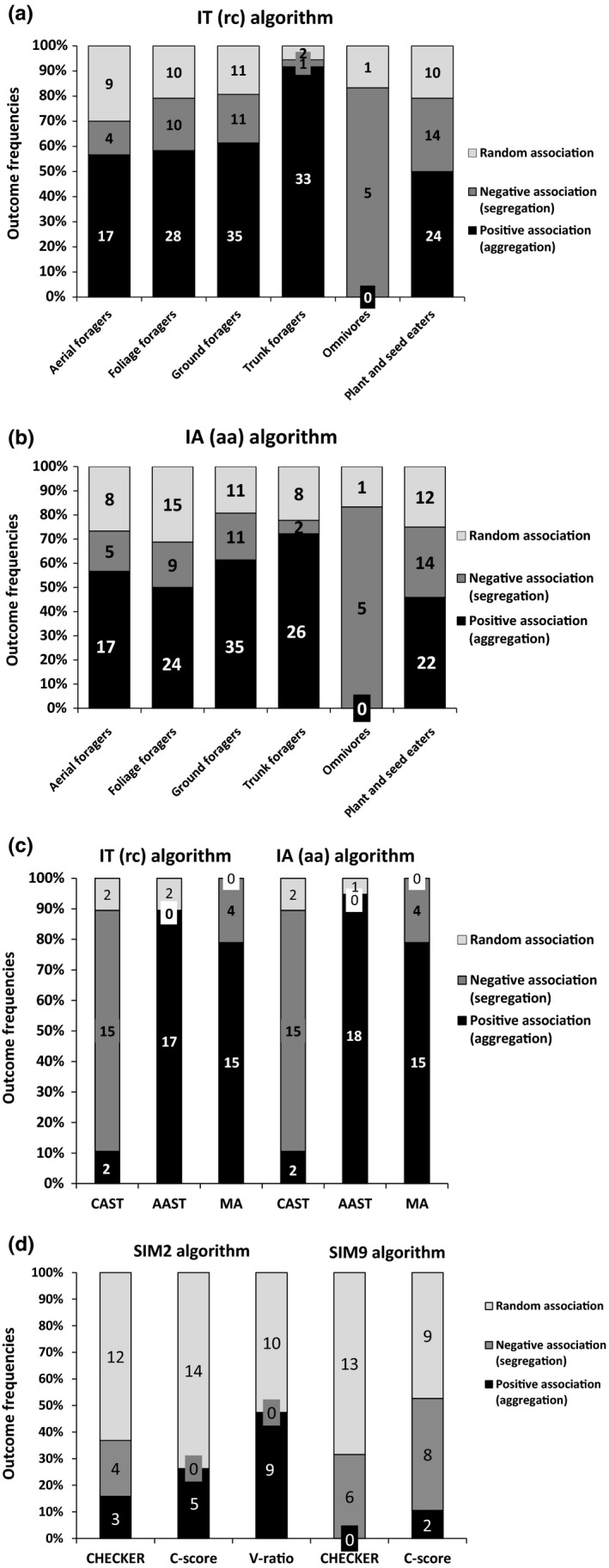
Summary of null model results testing for nonrandom patterns of species associations using quantitative (a‐c) or binary (d) matrices at the level of guild (a, b) and entire assemblages (c‐d). Association patterns are derived from temporal series of 19 breeding bird assemblages from Europe and North America

The effects of the characteristics of the data sets (see Preparation of data matrices) on the null model outcomes were analyzed using binomial and multinomial models. Because the frequencies in the outcome categories were unbalanced and the data set characteristics were intercorrelated, we fit each variable separately rather than combining them into one full model. When the null model simulation revealed dichotomous outcomes (cf. Figure 1), binomial Bayesian MCMC generalized linear models (GLM) with a logit link function were fitted to the data. In the case of trichotomous outcomes, the data were fitted with multinomial Bayesian GLMs. The priors and MCMC properties of the GLMs were specified in analogy to the GLMMs. The analyses were performed in R language version 3.5.0 (R Core Team, 2018) using the library MCMCglmm (Hadfield, 2010).

## 3 RESULTS

### 3.1 Patterns of species association in whole assemblages and guilds

We assessed the species association patterns in 19 different bird assemblages using four null model algorithms in combination with six association indices (Appendices [Link], [Link], [Link], [Link]). Combined results for the assemblages of the different null models revealed 42% with aggregated patterns and 31% with random patterns, and only 27% of null model simulations showed significantly segregated patterns in these bird assemblages. Quantitative null models showed aggregations more often (61%) than binary models (20%), while the pattern was almost opposite for the random outcomes (quantitative models = 6%, binary models = 61%; Figure 1). Both model types yielded slightly different amounts of segregated results: quantitative = 33.33% and binary models = 18.95%. Interestingly, binary null models with row sums fixed and column equiprobable constraint (SIM2) in combination with C‐score and V‐ratio did not reveal any segregated patterns in contrast to the SIM9 algorithm, which showed eight segregations with C‐score (Figure 1). For quantitative models, the proportion of random (5%–7%), positive (~60%), and negative associations (~33%) were similar regardless of whether the IT (rc) and IA (aa) algorithm was used.

Analyses on guild levels revealed similar association geometry (type; Appendix S7). Pooled results for both algorithms and co‐occurrence indices showed a strong prevalence of positive associations (~58%) and roughly similar frequencies of negative (~20%) and random associations (~22%). Positive associations strongly dominated in all guild types (~48%–82%) except omnivores (0%). Negative associations were represented in all guilds in less than 30% frequency except omnivores, in which segregation strongly dominated (~83%).

**Figure 2:**
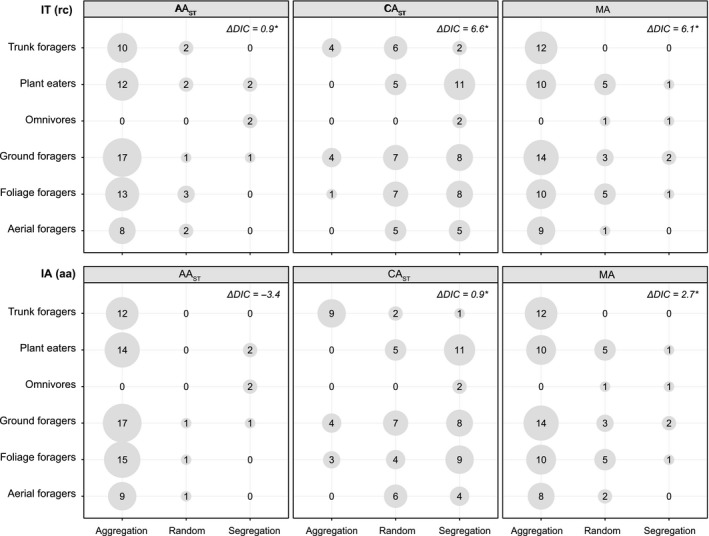
Differences in the null model outcomes among guilds as revealed by the combinations of algorithms IT (rc) and IA (aa) and three indices (AA_ST_, CA_ST_, and MA). The results of the Bayesian GLMMs are summarized as differences in deviance information criteria of each model and the associated null model (ΔDIC). Significantly different patterns among guilds are designated with asterisks. Circle sizes are proportional to the number of outcomes in each category

We used Bayesian GLMMs to analyze relationships between the category of guild and the outcome of null model simulation and found significant differences among guilds in all but one combination of index and algorithm (Figure 2). The differences among guilds were caused mainly by a significantly higher probability of aggregation in trunk foragers (indexes CA_ST_ and MA) and a higher tendency of segregation in omnivores (index AA_ST_) than in the other guilds. Patterns revealed by the GLMMs were similar for both null model algorithms.

### 3.2 The relationships between data set properties and the results of null model simulations

We used Bayesian GLMs to test the relationships between various data set characteristics and the proportion of the null model outcome categories (aggregation, segregation, and random). In general, the outcomes of both the binary and quantitative null models of whole assemblages were significantly related to the size of the census plot, while the outcomes of the quantitative models were consistently related to species density characteristics (Table 1).

**Table 1:** Results of the binomial and multinomial Bayesian GLMs analyzing relationships between the outcomes of the null model simulations (aggregation, segregation, and random) and various characteristics of the assemblage data sets

	Binary null models					Quantitative null models					
	SIM2 algorithm			SIM9 algorithm		IT (rc) algorithm			IA (aa) algorithm		
	CHECKER	C‐score	V‐ratio	CHECKER	C‐score	CA_ST_	AA_ST_	MA	CA_ST_	AA_ST_	MA
Plot size	−0.78	**3.51**	−0.15	**3.35**	**7.87**	0.30	**6.82**	**9.71**	0.27	**1.70**	**9.70**
Matrix size	0.28	−0.20	−1.14	**7.68**	−0.79	0.64	−1.45	−0.87	0.57	−0.95	−0.87
Number of years	−0.03	0.46	−0.39	**7.71**	−0.79	0.72	−0.27	2.68	0.72	0.48	2.66
Number of species	−0.35	−0.85	−1.19	2.12	0.13	−0.40	−0.60	−1.13	−0.45	−0.97	−1.14
Number of zeros	0.59	−1.02	−0.62	0.40	0.07	0.71	−0.93	−0.89	0.70	−1.03	−0.89
Mean density	–	–	–	–	–	**5.40**	**6.34**	**3.67**	**5.36**	−1.39	**3.66**
CV of density	–	–	–	–	–	−0.21	0.61	−0.08	−0.22	1.04	−0.09

Note

Differences in deviance information criteria of each model and the associated null model (ΔDIC) are displayed. Statistically significant results are highlighted in bold.

**Figure 3:**
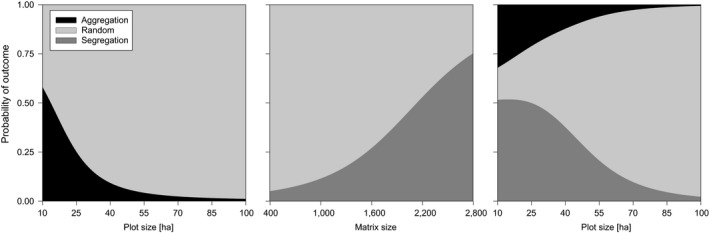
Examples of fitted probabilities (means of posterior distributions) from the significant binomial and multinomial GLMs testing the effect of plot size (C‐score, SIM2 algorithm), matrix size (CHECKER, SIM9 algorithm), and plot size (C‐score, SIM9 algorithm) on the outcomes of the binary null models

In binary models, the probability of detecting random patterns increased with the size of the census plot. On the other hand, matrix size and length of study positively affected the proportion of the segregated outcomes. Three examples of these patterns are displayed in Figure 3, but the same tendencies can be seen in each binary null model regardless of the type of co‐occurrence index.

**Figure 4:**
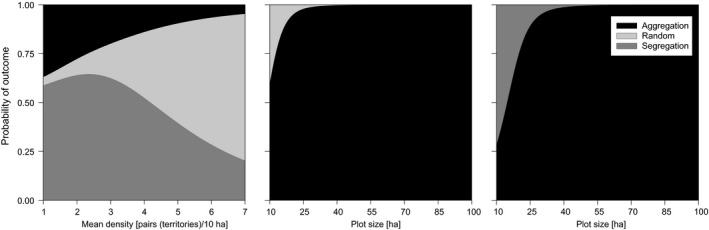
Examples of fitted probabilities (means of posterior distributions) from the significant binomial and multinomial GLMs testing the effects of mean species density (CA_ST_) and plot size (AA_ST_ and MA) on the outcomes of quantitative null models using the IT (rc) algorithm

In quantitative models of whole assemblages, the probability of detecting aggregated patterns in the bird assemblages increased steeply with the study plot size, while the proportion of random associations (CA_ST_ and AA_ST_) and segregations (MA) increased with the density of pairs (Figure 4).

Considering guild data sets, only 9% of GLMs showed a significant relationship with matrix properties on the null model outcomes (Appendix S8). In general, plot size, matrix size, number of species, and density of pairs were the main correlates of the null model outcomes. Plot size was positively linked with the probability of aggregations in aerial foragers, foliage foragers, ground foragers, and plant eaters. The probability of nonrandom associations significantly increased with matrix size in aerial and trunk foragers (aggregations) and ground foragers and plant eaters (segregations). Higher species richness in the guilds of aerial and trunk foragers led to a significantly higher proportion of aggregations, while ground foragers were significantly more segregated. Finally, a higher density of breeding pairs was associated with an increased probability of random association in ground foragers and elevated segregations in plant eaters.

## 4 DISCUSSION

### 4.1 Patterns of species association in assemblages

Results of the current and our previous study (Korňan & Svitok, 2018) of the 19 long‐term bird data sets showed a strong prevalence of positive and random associations, which indicate prevalence of correlated and random dynamics driving the temporal patterns of bird assemblages. These findings do not support the assumption of competition theory based on compensatory dynamics. On the other hand, our results agree with the conclusions of Houlahan et al. (2007) who concluded that compensatory dynamics are rare in natural ecological communities. The predominance of correlated dynamics in temporal patterns may indicate a similar response of the majority of species to climate or stochastic factors. Hallet et al. (2014) evaluated the importance of several biotic mechanisms (compensatory dynamics, portfolio effects, the selection for stable dominant species) on the stability of species abundance and richness of plant communities in connection with two key environmental factors, precipitation amount and its variability. They concluded that all the mentioned mechanisms may play an important role and that their importance could vary across environmental gradients. This study suggests that the dynamics of ecological communities may depend on several biotic drivers that can predominate under different climatic and stochastic scenarios.

In contrast to temporal data, spatial data sets show strong predominance of negative species association and, thus, support for the concept of checkerboard (complementary) distribution of organisms (Diamond, 1975) that is one of the most widely tested concepts in ecology (Gotelli & McCabe, 2002). Several empirical studies based on large published data sets of various taxonomic groups analyzed by binary or quantitative null models support this concept (Gotelli & McCabe, 2002; Gotelli & Ulrich, 2010; Ulrich & Gotelli, 2010). In addition, other taxon‐specific studies provide evidence that negative species associations may drive spatial patterns of co‐occurrence in some biological communities (e.g., Abu Baker & Patterson, 2011; Badano, Regidor, Núñez, Acosta, & Gianoli, 2005; Heino & Soininen, 2005; Kobza, Trexler, Loftus, & Perry, 2004; Sarà, Bellia, & Milazzo, 2006). On the other hand, there also exist opposite opinions supported by similar meta‐analyses and other taxon‐specific studies indicating predominance of positive or random association in communities in spatial patterns (e.g., Connor, Collins, & Simberloff, 2013; Feeley, 2003; Götzenberger et al., 2012; Jenkins, 2006; Korňan & Korňan, 2016; Perez‐Neto, 2004; Pitta, Giokas, & Sfenthourakis, 2012; Šálek, Červinka, Padyšáková, & Kreisinger, 2014; Schluter, 1984; Sfenthourakis, Tzanatos, & Giokas, 2006; Wang, Chen, & Ding, 2011). The dominant role of environmental drivers and lack of generality of competition‐driven mechanism in elevational species range replacement were described in elevational studies of birds (e.g., Bastianelli, Wintle, Martin, Soane, & Laiolo, 2017; Elsen, Tingley, Kalyanaraman, Ramesh, & Wilcove, 2017) that support our conclusion that competition does not seem to be main factor driving temporal bird assemblage patterns. Mönkkönen, Devictor, Forsman, Lehikoinen, and Elo (2017) suggest that community assemblies on a local scale can reflect both positive associations from social information use among species (heterospecific attractivity) as well as negative associations coming from competitive interactions, habitat filters, and dispersal abilities.

Negative species association and missing species combinations have mainly been interpreted as results of competitive species interactions or environmental filters (see Mayfield & Levine, 2010 for competition relatedness hypothesis); however, these same patterns may be generated by unique habitat associations (Perez‐Neto, Olden, & Jackson, 2001), limited dispersal (Ulrich, 2004), and historical or evolutionary processes that prevent species co‐occurrence without the possibility of interspecific interactions (Bloch, Higgins, & Willing, 2007; Ulrich & Gotelli, 2007). In addition, based on a modelling approach, Ulrich, Jabot, and Gotelli (2017) concluded that under neutral dispersal, competitive interactions may change the geometry of species associations. This means that species association analysis alone is not capable of drawing simple conclusions about the effects of competitive interactions on patterns of co‐occurrence. Ulrich, Kryszewski, et al. (2017) proposed a comprehensive triangular framework to statistically distinguish and evaluate patterns of segregation (turnover), modularity, and nestedness. It seems to be a promising approach to evaluate and disentangle complex community matrices into basic functional trait systems that distinguish competition from habitat filtering.

The contrasting results of studies using different data sets in the search for assembly rules (compensatory dynamics and complementary distribution) could be at least partly related to differences in methodology and environmental settings that can mimic or obscure competitive interactions. Both sources of potential confusion are briefly discussed below and exemplified in our study of bird assemblages.

### 4.2 The effect of spatial scale

The importance of spatial scale in ecology has long been recognized (Wiens, 1989b). It is clear that competitive interactions are scale‐dependent and that the outcomes of null model simulations are related to the spatial extent of the study. Assembly rules are expected to be mainly apparent at relatively small spatial scales, but the specific spatial extent at which assembly rules act largely depends on the group studied (Götzenberger et al., 2012; Wilson & Stubbs, 2012). In the bird assemblages investigated here, we found significant support for the effect of study area size on the outcomes of null models. The probability of detecting random patterns increased with census plot size in binary models, which is consistent with the above mentioned expectations. Interspecific competition among competing bird species is expected on a small spatial scale, in which competitively superior species exclude competitively inferior species in the years of their presence. On larger spatial scales, this does not necessarily apply because in large plots, there is a higher probability of spatial segregation of the territories of competing species; thus, competitive exclusion does not have to be reflected in the presence/absence data patterns. In quantitative analyses on both assemblage and guild levels (aerial foragers, foliage foragers, ground foragers, and plant eaters), we found a positive relationship between census plot size and the probability of aggregation. If most species within assemblages have common response to climate and stochastic factors as we assume, thus, increasing area of plot size also increases the probability of occurrence of more species; thus, the effects of aggregation may hold stronger. Indeed, increasing spatial scale increases habitat heterogeneity, and the random patterns observed in studies conducted over a larger scale may result from environmental control that obscures interspecific competition (Wilson, 1999). For example, during years of high productivity, bird species that are ecologically similar may coexist in the same sites (Cody, 1999), or simple availability of suitable habitats may allow for effective segregation of potential competitors at larger spatial scales, which may cause aggregations.

### 4.3 The effect of data types

Further discrepancies may arise from using data having different information content. For example, the analyses of bird assemblages provided here showed that many random patterns revealed in the presence/absence data become aggregations when quantitative information was included (Appendices [Link], [Link], [Link], [Link]). In general, quantitative data offer richer information than binary data and may therefore be more sensitive to relatively subtle changes in population size that precede competitive exclusion (Ulrich & Gotelli, 2010). However, direct comparison between binary and quantitative model outcomes is not straightforward, and researchers must be rather cautious, since abundance data are based on counts of individuals, not species counts, and both data types use different randomization algorithms in null model simulations (Gotelli & Ulrich, 2012). We used complete sets of species in the assemblages in both binary and quantitative analyses, and the results of both analyses showed a strong prevalence of random and positive associations. In fact, the high frequency of random associations (~61%) and the lower frequency of aggregations (20%) in binary analyses have predominantly shifted toward positive association in quantitative analyses. As we found, this happened in 58.3% and 61.1% of cases of random association classified by SIM2 algorithm compared to quantitative analyses carried out IT (aa) and IA (rc) algorithm. Moreover, we also used a subset of species in the assemblage matrices with measurable abundances (excluding rare species) in quantitative analyses, and these analyses also yielded similar patterns (results upon request from corresponding author). The exclusion of rare species increased the fill in the quantitative matrices (the average proportion of zeroes dropped from 38% to 2%). The reduced analyses showed approximately 54% aggregations, 26% random associations, and 20% segregations when pooling data for all null model and index combinations, yet this analysis was done only on 18 bird assemblages.

Another source of bias lies in disregarding the functional structure of assemblages, and consequently, null model analyses at the whole community level may suffer from dilution effect (Diamond & Gilpin, 1982). Most meta‐analyses of species associations conducted at the community level could have been influenced by the dilution effect (e.g., Gotelli & McCabe, 2002; Schluter, 1984; Ulrich & Gotelli, 2010). Gotelli and McCabe (2002) and Ulrich and Gotelli (2010) recognized the importance of the dilution effect, and even when they accounted for this effect, negative species associations prevailed in their results. We excluded the potential of dilution bias by using quantitative null model analyses on the guild levels in all assemblages. The results of the guild analyses were consistent with the assemblage‐wide analyses except for the guild of omnivores. We did not find reasonable explanation why species of this guild showed high level of segregation. Other bird foraging guild studies based on an *a posteriori* approach (Holmes et al., 1986; Korňan, 2013) detected positive species associations in two long‐term data sets of breeding bird assemblages in North America and Europe.

### 4.4 The effects of null model settings

When addressing nonrandom patterns in community matrices, a crucial step is the choice of the species association index and the null model algorithm (Gotelli & Graves, 1996). In general, many combinations of association indices with null model algorithms are possible, each of which differs in its statistical properties and ecological assumptions (Gotelli, 2000; Ulrich & Gotelli, 2010). Even a relatively trivial choice between proven and widely used indices may lead to different results. In our analyses, for example, the variance ratio index and C‐score showed a higher tendency for the detection of aggregation patterns than the number of checkerboard species pairs (cf. Figure 1). In contrast to the CHECKER index, the other two co‐occurrence indices do not directly measure co‐occurrence but rather some aspect of its manifestation. Specifically, the variance ratio measures variability in the total number of species in an assemblage, and the C‐score is the mean number of checkerboard units per species pair. Consequently, the choice of co‐occurrence indices may lead to different conclusions regarding nonrandom assembly patterns. For example, Gotelli and McCabe (2002), analyzing evidence for Diamond's assembly rules, argued that the different conclusions of their study and that of Schluter (1984) could be attributable to the different co‐occurrence indices used.

The choice of the null model algorithm can affect the results of the analysis even more profoundly than the choice of the association index. For example, Ulrich and Gotelli (2010) argued that the prevalence of positive associations in the studies of Schluter (1984) and Houlahan et al. (2007) could be a result of inadequate assumptions of equivalence among sites and time samples. They noted that the correct approach would be to use the IT algorithm that preserves the column totals, allowing for differences among sites or time samples. In a recent meta‐analysis of published data, Götzenberger et al. (2012) found that co‐occurrence patterns of plant assemblages significantly differed between models that keep species frequencies over the plots and richness within the plots constant (SIM9) and those that kept only the species frequencies constant (SIM2). In general, their SIM2 models showed aggregated patterns, while SIM9 models tended to segregate outputs. This corresponds with our results, where models with the SIM9 algorithm did not show any aggregated pattern. A slight bias toward detecting segregated co‐occurrence with the SIM9 algorithm and C‐score index is already known (Ulrich & Gotelli, 2007). If only the C‐score and the SIM9 model had been used here, the overall picture would change, and segregated patterns would prevail (42%). Although the SIM9 model has good statistical properties, its use with a C‐score is not recommended for matrices that are highly filled, such as those analyzed here (mean fill = 61%). In these cases, the more conservative indices, such as the number of checkerboards, appear to be more appropriate (Ulrich & Gotelli, 2007). Adopting this approach would lead to the dominance of random associations (68%) in the studied assemblages.

Finally, the difference in the ability of null models to reveal a nonrandom pattern when it truly exists (statistical power) should be briefly discussed. Gotelli and Ulrich (2012) pointed out that null model analysis may not be well suited for large data sets, since large data sets may often deviate significantly from null models regardless of whether species occurrences are random. Considering the bird data sets, the relationships between the matrix size and the statistical power of the analysis were particularly apparent in the binary models (Table 1). For example, when the matrix size increased above approximately 2,200, null models with the CHECKER index and SIM9 algorithm had the tendency to reveal predominantly segregated patterns in bird assemblages (Figure 3). Similarly, Gotelli and McCabe (2002) found that matrix size was significantly related to null model outputs because large matrices enhanced the statistical power of the analysis. These two examples suggest that the issue of statistical power must be kept in mind when comparing results of different null model studies.

Based on the latest finding of Ulrich et al. (2018), most null model algorithms are prone to the effects of total matrix species richness on association patterns. Only the fixed–fixed algorithm (SIM9) showed a weak correlation with total matrix species richness, and it is strongly recommended for species association studies. In our study, we used this algorithm in combination with the C‐score metric, as it is recommended in Ulrich et al. (2018), and with other two metrics. We also used the SIM2 algorithm, which has a reasonable ecological basis but can be prone to the effects of total matrix species richness. Ulrich et al. (2018) used proportional–proportional and equiprobable–equiprobable algorithms that had a tendency toward segregations and aggregations. Even though, Ulrich et al. (2018) did not provide the results of the simulation for SIM2 (fixed–equiprobable algorithm), we interpreted the results of the null model simulation by SIM2 with caution in this manner.

### 4.5 Confounding effect of environmental variability

It should be clearly stated that null models based on data sets consisting of field observations are by no means definitive tests of the importance of competitive interactions, since the same nonrandom patterns can be explained by other mechanisms. For example, species segregation can also arise from habitat variability, while aggregation might be caused by interspecific facilitation (Gotelli & McCabe, 2002; Götzenberger et al., 2012; Wilson, 1999). Considering bird assemblages, many species are known to prefer specific optimal microhabitats or successional stages; consequently, the spatial distribution of these species is nonrandom and is strongly affected by forest heterogeneity. The dominant habitat types investigated here were natural and primaeval forests characterized by high horizontal and vertical heterogeneity formed by natural tree falls (gaps), local successions, patches of different developmental stages, uprooted trees, and multi‐storey profiles of stands (Korňan, 2013; Wesołowski, 2007). This patchy environment in combination with the different microhabitat requirements of species may cause clumped habitat occupancy patterns at larger scales. This phenomenon could cause patterns with high species richness at the census plot level that are transferred to highly filled matrices, which bias the results of null model analyses toward random or positive species associations. Therefore, using binary matrices from relatively large census plots (≥ 10 ha) does not necessarily yield competition patterns, which could be more probable from point samples. Nevertheless, this can be overcome by using quantitative matrices with population density data, as interactions are estimated by changes in abundance on the plot level. This is essentially because larger plots are needed for objectively estimating abundance or population density in forest habitats (minimum plot size for forest habitats in the mapping method is 10 ha) that may better reflect overall differences in the quantitative assemblage structure between years in a studied habitat. This study used both binary and quantitative data, but neither led to the prevalence of segregations in the analyses. In fact, binary data analyses led to the prevalence of random associations, whereas the quantitative data analyses led to the prevalence of aggregations. In summary, the results do not underscore compensatory dynamics processes as the main drivers of bird assemblage dynamics.

## CONFLICT OF INTEREST

None declared.

## AUTHORS' CONTRIBUTIONS

MK developed the ideas and designed methodology; MK collected the data from one site; MK and AK classified birds into guilds; MK and MS performed statistical analyses, MK led the writing of manuscript in co‐operation with MS and AK. All authors contributed critically to the drafts and gave final approval for publication.

## Data Availability

Raw meta‐data supporting information in Appendix S1 is available in Dryad Digital Repository https://doi.org/10.5061/dryad.bd6m135.
