# Supplementary material for: Null model analyses of temporal patterns of bird assemblages and their foraging guilds revealed the predominance of positive and random associations
Source: Ecol Evol. 2019 Jun 20;9(15):8541–54. doi: 10.1002/ece3.5372 (PMC6686305; doi:10.1002/ece3.5372)
Supplement: Supplementary file 1 [file ECE3-9-8541-s001.docx]

Supplement 1.  List of analyzed breeding bird assemblages by binary and quantitative null models from various habitat types in Europe and North America, where mapping method was applied for population estimates. Fifteen study sites represented forests, three study site open habitats and one study site a city park. The citations of studies from which the published data sets were used for the analyses are given as superscripts in the column „Study site“. The numbers in parentheses in the time period column indicate the number of study years. The matrix fill indicates the proportion of non-zero values in the data matrices.

| **Study site** | **Location** | **Habitat type** | **Time period** | **Plot size (ha)** | **Number of analyzed species** | **Number of analyzed guilds** | **Matrix fill** |
| --- | --- | --- | --- | --- | --- | --- | --- |
| Šrámková National Nature Reserve^1^ | Europe, Slovakia | primeval beech-fir forest | 1997-2006 (10) | 27.5 | 53 | 5 | 73.77 |
| Białowieża National Park, plot CM^2^ | Europe, Poland | primeval oak-lime-hornbeam forest | 1975-2014 (40) | 24 | 52 | 5 | 63.80 |
| Białowieża National Park, plot K^2^ | Europe, Poland | primeval riverine alder-ash-spruce forest | 1975-2014 (40) | 33 | 78 | 5 | 61.70 |
| Białowieża National Park, plot L^2^ | Europe, Poland | primeval alder swamp | 1976-2014 (37)^13^ | 25 | 60 | 5 | 61.58 |
| Białowieża National Park, plot MS^2^ | Europe, Poland | primeval oak-lime-hornbeam forest | 1975-2014 (40) | 30 | 53 | 5 | 60.47 |
| Białowieża National Park, plot NE^2^ | Europe, Poland | primeval spruce-pine-birch forest | 1975-2014 (40) | 25 | 55 | 5 | 58.86 |
| Białowieża National Park, plot NW^2^ | Europe, Poland | primeval spruce-pine-birch forest | 1975-2014 (40) | 25 | 56 | 5 | 57.95 |
| Białowieża National Park, plot W^2^ | Europe, Poland | primeval oak-lime-hornbeam forest | 1975-2014 (40) | 25.5 | 69 | 5 | 57.46 |
| Słowacki park, City of Wrocław^3^ | Europe, Poland | city park | 1970-1999 (29) | 7-7.5 | 35 | 5 | 68.77 |
| Gaisatjakke and Valle Mts^4^ | Europe, Sweden | primeval subalpine birch forest | 1963-1999 (37) | 36.2-62.2^19^ | 35 | 3 | 49.03 |
| Estenstad forest^5^ | Europe, Norway | secondary spruce forest | 1960-1972 (12)^14^ | 100 | 34 | 3 | 80.15 |
| Birdsong valley^6^ | Europe, Sweden | isolated secondary oak-ash forest | 1953-2009 (57) | 13 | 41 | 3 | 51.73 |
| Dalby Söderskog National Park^7^ | Europe, Sweden | secondary ash-elm-oak-beech forest | 1980-1993 (13)^15^ | 37 | 43 | 4 | 67.98 |
| Ammarnäs region, plot K1^8^ | Europe, Sweden | alpine dry scrub heath and mires | 1964-1983 (20) | 100 | 22 | 2 | 52.73 |
| Ammarnäs region, plot K2^8^ | Europe, Sweden | alpine dry scrub heath and mires | 1964-1983 (20) | 100 | 17 | 1 | 47.06 |
| Finsefetene sedimentation flat^9^ | Europe, Norway | Intermediate fens, water bodies, silt dunes | 1967-1984 (18)^16^ | 100 | 14 | 1 | 59.24 |
| Bookham Common^10^ | Europe, England | isolated secondary oak forest | 1950-1975 (25)^17^ | 16.19 | 44 | 4 | 71.36 |
| Hubbard Brook^11^ | North America, New Hampshire | secondary beech-maple-birch forest | 1969-2013 (45) | 10 | 33 | 3 | 67.41 |
| William Trelease Woods^12^ | North America, Illinois | isolated original maple-hackberry-ash forest | 1927-1976 (44)^18^ | 24 | 61 | 6 | 45.38 |

1 Korňan 2013

2 Abundance data for the whole study period are given is studies of Tomiałojć et al. 1984, Tomiałojć & Wesołowski 1994, Tomiałojć & Wesołowski 1996, Wesołowski et al. 2002, Wesołowski et al. 2006, Wesołowski et al. 2010, Wesołowski et al. 2015.

3 Tomiałojć 2011

4 Enemar et al. 2004 (Densities from the period 1963−1982 are also given in Enemar et al. (1984), however the numbers are not identical.)

5 Hogstad 1993

6 Svensson et al. 2010

7 Svensson 2009

8 Svensson et al. 1984

9 Abundance data on passerines are published in Østbye et al. (2002) and abundance data on waders and gulls are from Østbye et al. (2007).

10 Beven 1976

11 Abundance data provided by Richard T. Holmes for the whole study period, partial data set is published in Holmes et al. 1986 (1969-1984) and on the Internet site <http://www.hubbardbrook.org/data/dataset_search.php> (1969-2004).

12 Kendeigh 1982

13 Years 1975, 1978 and 1979 are missing.

14 Year 1961 is missing.

15 Year 1981 is missing, *Sturnus vulgaris* excluded from the analyses due to irregular censusing.

16 Passerines were not censused in 1982 and this year is excluded from analyses.

17 Year 1949 was not included to the data set because species *Cyanistes caeruleus* and *Prunella modularis* were not censused in this year.

18 Period 1929‒1933 and year 1938 are missing.

19 Data published in the paper represent pooled data from six study plots (8‒12.8 ha each) censused in the study period. The total area of the censused study plots in a year varied as indicated.

References

Beven, G. (1976). Changes in breeding bird populations of an oak-wood on Bookham Common, Surrey, over twenty-seven years. *London Naturalist,* 55, 23–42.

Enemar, A., Nilsson, L., & Sjöstrand, B. (1984). The composition and dynamics of the passerine bird community in a subalpine birch forest, Swedish Lapland. A 20-year study. *Annales Zoologici Fennici,* 21, 321–338.

Enemar, A., Sjöstrand, B., Anderson, G., & von Proschwitz, T. (2004). The 37-year dynamics of a subalpine passerine bird community, with special emphasis on the influence of environmental temperature and *Epirrita autumnata* cycles. *Ornis Svecica,* 14, 63−106.

Holmes, R.T., Sherry, T.W., & Sturges, F.W. (1986). Bird community dynamics in a temperate deciduous forest: long-term trends at Hubbard Brook. *Ecological Monographs,* 56, 201–220.

Hogstad, O. (1993). Structure and dynamics of passerine bird community in a spruce-dominated boreal forest. A 12-year study. *Annales Zoologici Fennici,* 30, 43–54.

Kendeigh, S.C. (1982). Bird populations in east central Illinois: fluctuations, variations, and development over a half-century. *Illinois Biological Monographs,* 52, 1–137

Korňan, M. (2013). Breeding bird assemblage dynamics of a primaeval temperate mixed forest in the Western Carpathians (Slovakia): support for pluralistic community concept. *Ornis Fennica,* 90, 151–177.

Østbye, E., Hogstad, O., Østbye, K., Lien, L., & Framstad E. (2002). Structure and dynamics of some high mountain bird communities of South Norway: a 19-year study of passerines. *Ornis Norvegica,* 25, 19−48.

Østbye, E., Hogstad, O., Østbye, K., Lien, L., Framstad E., & Breiehagen T. (2007). Structure and dynamics of a high mountain wetland bird community in southern Norway: An 18-year study of waders and gulls. *Ornis Norvegica,* 30, 4−20.

Svensson, S. (2009). A stable bird community during 27 years (1980–2006) in the nemoral broadleaf wood Dalby Söderskog National Park. *Ornis Svecica,* 19, 237–243.

Svensson, S., Carlsson, U.T., & Liljedahl, G. (1984). Structure and dynamics of an alpine bird community, a 20-year study. *Annales Zoologici Fennici,* 21, 339–350.

Svensson, S., Thorner, A.M., & Nyholm, N.E. (2010). Species trends, turnover and composition of a woodland bird community in southern Sweden during a period of fifty-seven years. *Ornis Svecica,* 20, 31–44.

Tomiałojć, L. (2011). Changes in breeding bird communities of two urban parks in Wrocław across 40 years (1970–2010): before and after colonization by important predators. *Ornis Polonica*, 52,1-25.

Tomiałojć, L., & Wesołowski, T. (1994). Die Stabilität der Vogelgemeinschaft in einem Urwald der gemässigten Zone: Ergebnisse einer 15jährigen Studie aus dem Nationalpark von Białowieża (Polen). *Ornithologische Beobachter,* 91, 73–110.

Tomiałojć, L., & Wesołowski, T. (1996). Structure of a primaeval forest bird community during 1970s and 1990s (Białowieża National Park, Poland). *Acta Ornithologica,* 31, 133–154.

Tomiałojć, L., Wesołowski, T., & Walankiewicz, W. (1984). Breeding bird community of a primaeval temperate forest (Białowieża National Park, Poland). *Acta Ornithologica,* 20, 241–310.

Wesołowski, T., Czeszczewik, D., Hebda, G., Maziarz, M., Mitrus, C., & Rowiński, P. (2015). 40 years of breeding bird community dynamics in a primeval temperate forest (Białowieża National Park, Poland). *Acta Ornithologica*, 50, 95−120.

Wesołowski, T., Mitrus, C., Czeszczewik, D. & Rowiński, P. (2010). Breeding bird dynamics in a primeval temperate forest over thirty-five years: variation and stability in the changing world. *Acta Ornithologica,* 45, 209−232.

Wesołowski, T., Rowiński, P., Mitrus, C. &Czeszczewik, D. (2006). Breeding bird community of a primeval temperate forest (Białowieża National Park, Poland) at the beginning of the 21^st^ century. *Acta Ornithologica,* 41, 55–70.

Wesołowski, T., Tomiałojć, L., Mitrus, C., Rowiński, P., & Czeszczewik, D. (2002) The breeding bird community of a primaeval temperate forest (Białowieża National Park, Poland) at the end of the 20th century. *Acta Ornithologica,* 37, 27–45.
