# Supplementary material for: Null model analyses of temporal patterns of bird assemblages and their foraging guilds revealed the predominance of positive and random associations
Source: Ecol Evol. 2019 Jun 20;9(15):8541–54. doi: 10.1002/ece3.5372 (PMC6686305; doi:10.1002/ece3.5372)
Supplement: Supplementary file 6 [file ECE3-9-8541-s006.docx]

Supplement 6.  Results of quantitative null model analyses by the IA (aa) algorithm with three indices (CA – number of quantitative checkerboards, AA – number of quantitative aggregations, MA - Chaoʼs index of similarity of *n* communities) of 19 breeding bird assemblages from Europe and North America (see Table 1 for details). Observed values of individual indices, their simulated values (10 000 iterations), statistical probability (one-tailed test), and the types of detected species associations are given. The abbreviations of the algorithms and indices given in parenthesis follow Turnover 1.1 manual (Ulrich 2010).

| **Site/index** | **CA_ST_ (WCS)** |  |  | **AA_ST_ (Wtog)** |  |  | **MA (Chao)** |  |  | **Detected association** |  |  |
| --- | --- | --- | --- | --- | --- | --- | --- | --- | --- | --- | --- | --- |
|  | Observed | Simulated | P | Observed | Simulated | P | Observed | Simulated | P | **CA_ST_ (WCS)** | **AA_ST_ (Wtog)** | **MA (Chao)** |
| Ammarnäs region, plot K1 | 0.03 | 0.02 | 0.0002 | 0.15 | 0.03 | 0.0002 | 0.90 | 0.63 | 0.0002 | segregation | aggregation | aggregation |
| Ammarnäs region, plot K2 | 0.03 | 0.02 | 0.0002 | 0.15 | 0.02 | 0.0002 | 0.77 | 0.40 | 0.0002 | segregation | aggregation | aggregation |
| Białowieża National Park, plot CM | 0.05 | 0.04 | 0.0002 | 0.23 | 0.10 | 0.0002 | 0.92 | 0.86 | 0.0002 | segregation | aggregation | aggregation |
| Białowieża National Park, plot K | 0.05 | 0.03 | 0.0002 | 0.21 | 0.09 | 0.0002 | 0.90 | 0.86 | 0.0002 | segregation | aggregation | aggregation |
| Białowieża National Park, plot L | 0.04 | 0.04 | 0.0002 | 0.21 | 0.10 | 0.0002 | 0.93 | 0.84 | 0.0002 | segregation | aggregation | aggregation |
| Białowieża National Park, plot MS | 0.04 | 0.03 | 0.0002 | 0.22 | 0.09 | 0.0002 | 0.92 | 0.85 | 0.0002 | segregation | aggregation | aggregation |
| Białowieża National Park, plot NE | 0.06 | 0.04 | 0.0002 | 0.22 | 0.08 | 0.0002 | 0.89 | 0.80 | 0.0002 | segregation | aggregation | aggregation |
| Białowieża National Park, plot NW | 0.05 | 0.04 | 0.0002 | 0.22 | 0.08 | 0.0002 | 0.89 | 0.79 | 0.0002 | segregation | aggregation | aggregation |
| Białowieża National Park, plot W | 0.04 | 0.03 | 0.0002 | 0.19 | 0.08 | 0.0002 | 0.92 | 0.86 | 0.0002 | segregation | aggregation | aggregation |
| Birdsong valley | 0.06 | 0.05 | 0.0002 | 0.16 | 0.16 | 0.1292 | 0.84 | 0.86 | 0.0016 | segregation | random | segregation |
| Bookham Common | 0.06 | 0.07 | 0.0002 | 0.23 | 0.20 | 0.0002 | 0.90 | 0.87 | 0.0026 | aggregation | aggregation | aggregation |
| Dalby Söderskog National Park | 0.06 | 0.05 | 0.4086 | 0.26 | 0.16 | 0.0002 | 0.91 | 0.87 | 0.0068 | random | aggregation | aggregation |
| Estenstad forest | 0.03 | 0.06 | 0.0002 | 0.26 | 0.08 | 0.0002 | 0.94 | 0.51 | 0.0002 | aggregation | aggregation | aggregation |
| Finsefetene sedimentation flat | 0.05 | 0.02 | 0.0002 | 0.17 | 0.02 | 0.0002 | 0.74 | 0.21 | 0.0002 | segregation | aggregation | aggregation |
| Gaisatjakke and Valle Mts. | 0.04 | 0.02 | 0.0002 | 0.15 | 0.07 | 0.0002 | 0.87 | 0.85 | 0.0450 | segregation | aggregation | aggregation |
| Hubbard Brook | 0.09 | 0.06 | 0.0002 | 0.27 | 0.18 | 0.0002 | 0.74 | 0.81 | 0.0002 | segregation | aggregation | segregation |
| Słowacki park, City of Wrocław | 0.05 | 0.05 | 0.6628 | 0.25 | 0.24 | 0.0002 | 0.88 | 0.96 | 0.0002 | random | aggregation | segregation |
| Šrámková National Nature Reserve | 0.06 | 0.04 | 0.0002 | 0.25 | 0.09 | 0.0002 | 0.95 | 0.82 | 0.0002 | segregation | aggregation | aggregation |
| William Trelease Woods | 0.06 | 0.03 | 0.0002 | 0.14 | 0.07 | 0.0002 | 0.54 | 0.67 | 0.0002 | segregation | aggregation | segregation |
