# Supplementary material for: Null model analyses of temporal patterns of bird assemblages and their foraging guilds revealed the predominance of positive and random associations
Source: Ecol Evol. 2019 Jun 20;9(15):8541–54. doi: 10.1002/ece3.5372 (PMC6686305; doi:10.1002/ece3.5372)
Supplement: Supplementary file 8 [file ECE3-9-8541-s008.docx]

Supplement 8.  Results of binomial and multinomial Bayesian GLMs analyzing relationships between the outcomes of null model simulations (aggregation, segregation, random) and various characteristics of the guild datasets. Differences in deviance information criterions between each model and associated null model (ΔDIC) are displayed. Statistically significant results are highlighted in bold. Note that omnivores were excluded for the analyses due to the low frequency of occurrence. No tests were performed in the case of invariable null model outcomes (–).

|  | IT (rc) algorithm | | | |  | IA (aa) algorithm | | | |
| --- | --- | --- | --- | --- | --- | --- | --- | --- | --- |
| Dataset characteristics | CA_ST_ | AA_ST_ | MA |  | | | CA_ST_ | AA_ST_ | MA |
| Aerial foragers |  |  |  |  | | |  |  |  |
| Plot size | -0.56 | 1.60 | -0.72 |  | | | -0.60 | **4.98** | -0.44 |
| Matrix size | 3.83 | **7.48** | -0.73 |  | | | 2.35 | 1.11 | -0.48 |
| Number of years | 0.29 | **4.88** | -0.88 |  | | | 0.16 | -0.16 | -0.62 |
| Number of species | 3.68 | **4.75** | -0.69 |  | | | 1.62 | 2.17 | -0.55 |
| Number of zeros | 0.10 | -1.15 | -0.96 |  | | | 0.68 | -0.33 | 0.41 |
| Mean density | 1.26 | -0.88 | -0.85 |  | | | 1.81 | -0.73 | -0.38 |
| CV of density | 1.02 | 0.05 | -0.16 |  | | | 0.62 | -0.15 | 0.01 |
| Foliage foragers |  |  |  |  | | |  |  |  |
| Plot size | -0.35 | **7.13** | -0.58 |  | | | -1.35 | 1.31 | -0.57 |
| Matrix size | -0.09 | -1.24 | -0.42 |  | | | -1.19 | -0.46 | -0.43 |
| Number of years | 1.49 | -1.20 | -0.85 |  | | | -0.19 | 0.23 | -0.84 |
| Number of species | -0.42 | 1.02 | 1.44 |  | | | 0.19 | -0.41 | 1.42 |
| Number of zeros | 0.72 | -1.00 | -0.76 |  | | | -0.44 | -1.22 | -0.80 |
| Mean density | 0.60 | 2.32 | 2.25 |  | | | -0.64 | -0.22 | 2.26 |
| CV of density | -0.09 | **6.78** | -0.41 |  | | | -0.44 | 0.23 | -0.40 |
| Ground foragers |  |  |  |  | | |  |  |  |
| Plot size | **4.10** | 1.69 | **2.94** |  | | | **4.09** | 1.67 | **2.91** |
| Matrix size | **3.34** | 2.13 | -1.19 |  | | | **3.35** | 2.05 | -1.20 |
| Number of years | -0.71 | 0.87 | -0.46 |  | | | -0.72 | 0.84 | -0.50 |
| Number of species | 4.63 | -0.13 | -0.67 |  | | | **4.66** | -0.20 | -0.68 |
| Number of zeros | 1.46 | -0.21 | -0.59 |  | | | 1.47 | -0.26 | -0.64 |
| Mean density | **5.03** | -0.79 | **3.35** |  | | | **5.01** | -0.85 | 3.29 |
| CV of density | 0.20 | 0.07 | -0.52 |  | | | 0.20 | -0.02 | -0.56 |
| Plant eaters |  |  |  |  | | |  |  |  |
| Plot size | -0.73 | 0.66 | 0.33 |  | | | -0.75 | **7.99** | 0.36 |
| Matrix size | **8.13** | -1.06 | -1.13 |  | | | **8.12** | -0.72 | -1.16 |
| Number of years | **7.44** | -0.87 | 1.99 |  | | | **7.45** | -0.34 | 1.99 |
| Number of species | 0.38 | -0.73 | -0.81 |  | | | 0.39 | 2.53 | -0.83 |
| Number of zeros | -0.16 | -0.84 | -0.12 |  | | | -0.15 | -0.13 | -0.11 |
| Mean density | -1.30 | -1.23 | -0.83 |  | | | -1.30 | **4.19** | -0.89 |
| CV of density | 0.46 | -0.53 | -0.11 |  | | | 0.47 | -0.77 | -0.12 |
| Trunk foragers |  |  |  |  | | |  |  |  |
| Plot size | 0.33 | -1.00 | – |  | | | 0.43 | – | – |
| Matrix size | -0.18 | **5.03** | – |  | | | 0.08 | – | – |
| Number of years | < 0.01 | **2.87** | – |  | | | 0.27 | – | – |
| Number of species | -0.50 | **3.90** | – |  | | | 0.15 | – | – |
| Number of zeros | -1.10 | -1.13 | – |  | | | 0.76 | – | – |
| Mean density | -0.39 | 2.10 | – |  | | | -0.22 | – | – |
| CV of density | -0.05 | 1.12 | – |  | | | 0.33 | – | – |
